# Supplementary material for: WordCommentsAnalyzer: A windows software tool for qualitative research
Source: F1000Res. 2018 Sep 4;7:536. Originally published 2018 May 3. [Version 2] doi: 10.12688/f1000research.14819.2 (PMC6137411; doi:10.12688/f1000research.14819.2)
Supplement: Supplementary file 3 [file f1000research-7-17614-s0002.tgz › b2a79496-4ada-47c4-9682-a99ce867836d.zip!/Eight Iranian Health Journals/Journal of Knowledge and Health_36.docx]

© Journal of Knowledge and Health. This work is licensed under CC BY 4.0 (https://creativecommons.org/licenses/by/4.0/).

Volume & Issue: دوره 4، شماره 2: 1388

Title: اثر عصاره‌ي هيدروالكلي گل گلرنگ (Carthamus tinctorius) بر كاهش قندخون در موش‌هاي صحرايي ديابتي شده با آلوكسان

http://knh.shmu.ac.ir/index.php/site/article/view/170

مقدمه: استفاده از درمان‌هاي غيردارويي (گياهان دارويي) رويكرد جديدي در كنترل بيماري‌ ديابت است. هدف از مطالعه حاضر، بررسي اثر عصاره هيدروالكلي گل‌گلرنگ بر قند خون و ليپيد پروفايل در موش‌هاي صحرايي ديابتي می­باشد. مواد و روش­ها: در اين تحقيق 18 سر موش صحرايي نر با وزن متوسط 220 – 180گرم به­طور تصادفي در سه گروه شش تايي، كنترل غير ديابتي، كنترل ديابتي، ديابتي تيمار شده با عصاره هيدروالكلي گل‌گلرنگ به ميزان 200 ميلي‌گرم بر كيلوگرم وزن بدن تقسيم شدند و تزريقات به­صورت درون صفاقي انجام شد. موش‌هاي صحرايي براي 16 ساعت ناشتا بوده و سپس خون در لوله‌هاي هپارينه براي تعيين ميزان انسولين، قند، تري‌گليسريد و كلسترول جمع‌‌آوري شد. نتايج: نتايج حاكي از كاهش معنادار ميزان قند در گروه‌هاي تيمار شده با عصاره هيدروالكلي گل‌گلرنگ بود (05/0>P). هم­چنين در گروه‌ تيمار شده، ميزان كلسترول خون كاهش معنا‌دار و ميزان انسولين افزايش معناداري نسبت به گروه ديابتي يافته بود (05/0>P). آزمايشات بافت‌شناسي نشان دادند كه عصاره گل گلرنگ در موش‌هاي صحرايي ديابتي سبب بازسازي بافت پانكراس آسيب‌ديده شده است. نتيجه‌گيري: اين نتايج نشان مي‌دهند كه عصاره هيدروالكلي گل‌گلرنگ ممكن است در درمان ديابت مؤثر باشد. تأثير اين عصاره احتمالاً به دليل وجود فلاونوئيدها و خواص آنتي­اكسيداني آن­ها است.

Title: اثر ممانعت¬كنندگي عصاره سير بر رشد باكتري‌هاي سالمونلا تيفي موريوم و شيگلا ديسانتري

http://knh.shmu.ac.ir/index.php/site/article/view/171

مقدمه: امروزه استفاده از عصاره­های گیاهی به­عنوان افزودنی ضد میکروبی نقش مهمی در حفظ کیفیت مواد غذایی دارد. سیر از جمله گیاهانی است که با بررسی­های بیوشیمیایی تأثیرات ضد میکروبی آن به اثبات رسیده است. هدف از این تحقیق، ارزیابی اثر غلظت­های مختلف عصاره حاصل از پودر سیر و عصاره حاصل از قرص سیر در شرایط یکسان، بر باکتری­هاي سالمونلا تیفی موریوم و شيگلا ديسانتري مي­باشد. مواد و روش­ها: به­منظور انجام اين بررسی، سیر تازه از منطقه همدان و قرص سیر از شرکت داروسازی کوثر تهیه گردید. حداقل غلظت ممانعت­کنندگی و حداقل غلظت میکروب­کشی عصاره حاصل از پودر سیر و قرص سير بر رشد دو میکروارگانیسم به­روش استاندارد لوله­ای اندازه­گیری شد. نتایج: حداقل غلظت ممانعت­کنندگی، عصاره حاصل از پودر سیر بر میکروارگانیسم­های آزمایش شده 5/12 میلی­گرم بر میلی­لیتر و حداقل غلظت­کشندگی آن 25 میلی­گرم بر میلی­لیتر حاصل شد، درحالی­که حداقل غلظت ممانعت­کنندگی عصاره حاصل از قرص سیر بر رشد این میکروارگانیسم 40 میلی­گرم بر دسی­لیتر و حداقل غلظت­کشندگی آن 80 میلی­گرم بر دسی­لیتر به­دست آمد. طبق نتایج اخذ شده، عصاره سیر از رشد هر دو باکتری به­صورت يكسان ممانعت نموده، اما حداقل غلظت ممانعت­کنندگی عصاره حاصل از پودر سیر به مراتب قوی­تر از عصاره حاصل از قرص سیر می­باشد (3/3 برابر). نتیجه­گیری: با توجه به نتایج به­دست آمده از این بررسی و سایر گزارشات موجود در این زمینه، استفاده از این عصاره در سیستم­های نگهدارنده موادغذایی، برای جلوگیری از آلودگی­های میکروبی و بیماری­های ناشی از آن­ها مفید خواهد بود.

Title: بررسي علایم غيراختصاصي در ریفلاكس مري: مطالعه ی مبتني بر جمعيت

http://knh.shmu.ac.ir/index.php/site/article/view/175

مقدمه: ریفلاكس مري يك اختلال شايع دستگاه گوارش در سراسر جهان است. برخي از افراد مبتلا به ریفلاكس مري علامتي نداشته و يا داراي علایم غيراختصاصي مي­باشند. هدف از این مطالعه تعيين فراواني علایم غيراختصاصي و ارتباط آن با بيماري ریفلاكس مری می­باشد. مواد و روش­ها: اين مطالعه به­صورت مقطعي و مبتني بر جمعيت، بر روی 782 نفر كه به­صورت تصادفي ساده از 2 شهر فيروزكوه و دماوند در شمال استان تهران انتخاب شده بودند، طرح­ریزی و اجرا شده است. ارتباط ميان علایم غيراختصاصي و ریفلاكس با استفاده از آزمون كاي– اسكوئر مورد ارزيابي قرار گرفت و نسبت شانس با حدود اطمينان 95% براي هر يك از علایم محاسبه گرديد. نتايج: در این مطالعه 228 نفر (2/29%) مبتلا به ریفلاکس مری تشخیص داده شدند که شايع­ترين علامت غيراختصاصي در افراد تحت مطالعه، درد شكمي بود كه به­طور واضحي در خانم­ها بيش­تر مشاهده شد. احساس بغض در گلو، سرفه و تنگي نفس و بوي بد دهان از ديگر علایمي بودند كه ارتباط مستقيمي با ریفلاكس داشتند. ارتباط معناداري ميان سن و وجود علایم غير اختصاصي ریفلاكس مشاهده نگرديد. نتيجه­گيري: به­طور خلاصه، اين مطالعه نشان داد كه علایم غيراختصاصي ریفلاكس مري شايع بوده و ارتباط قوي با وجود اين بيماري دارد، بنابراين توجه به اين علایم در فرآيند تشخيص بيماري ریفلاكس مري، ضروري به­نظر می­رسد.

Title: بررسی تأثیر آموزش بهداشت فردی در پیش گیری از ابتلای مجدد به انگل های روده ای در دانش آموزان شهر گرگان

http://knh.shmu.ac.ir/index.php/site/article/view/174

مقدمه: کودکان به سبب پایین­تر بودن سطح ایمنی و تماس بیش­تر با خاک و مواد آلوده بیش­تر از بزرگسالان در معرض ابتلا به بیماری­های انگلی قرار دارند. این پژوهش با هدف تعیین اثر آموزش بهداشت فردی در پيشگیری از ابتلای مجدد به انگل­های روده­ای در دانش­آموزان شهر گرگان انجام شده است. مواد و روش­ها: در این مطالعه مداخله­ای دو مدرسه دخترانه ابتدایی شهر گرگان به­صورت خوشه­ای انتخاب و از ميان آن­ها 119 دانش­آموز به­صورت تصادفي انتخاب شدند و از آن­ها در سه نوبت آزمایش مدفوع گرفته و در آزمايشگاه با روش­هاي مستقيم، فلوتاسيون و فرمالين - اتر بررسي شدند. از 119 دانش­آموز، 40 نفرآلوده به انگل بودند. افراد مبتلا به 2 گروه به­صورت تصادفی تخصیص شدند. گروه مداخله علاوه بر دریافت درمان، آموزش­های لازم جهت نحوه انتقال انگل­ها و نیز روش­های پیش­گیری از آلودگي­هاي انگلي نیز دریافت نموده و گروه شاهد فقط درمان را دریافت نمودند. پس از دو ماه از هر دو گروه مجدداً نمونه مدفوع گرفته و آزمايش شدند. تجزیه و تحلیل با استفاده از آزمون­های کای- دو و برآورد نسبت خطر صورت پذیرفت. نتايج: از این 40 نفر، 3/24% آلوده به انگل­هاي بيماري­زا و 2/9% آلوده به انگل­هاي غير بيماري­زا بودند. در 35% از افراد گروه مداخله و 75% از افراد گروه شاهد پس از آموزش، ابتلای مجدد به آلودگی انگلی مشاهده شد. گروه مداخله با گروه شاهد از نظر میزان ابتلا به کرم­های انگلی اختلاف معناداری داشتند (7/4-0/1 CI:95/0, 1/2=,RR 02/0=P). نتيجه­گيري: نتايج اين مطالعه نشان داد كه آموزش بهداشت فردي خطر ابتلاي مجدد به انگل­های روده­ای را كاهش مي­دهد.

Title: ارتقاء کیفیت زندگی مردان سالمند کهریزک بر اساس مداخله آموزشی

http://knh.shmu.ac.ir/index.php/site/article/view/173

مقدمه: متوسط طول عمر در قرن اخیر در حال افزایش می­باشد و در حال حاضر، حدود 7% از جمعیت دنیا را افراد بالای 65 سال تشکیل می­دهند. تداوم و نگهداری سطح بالای کیفیت زندگی با افزایش سن از جمله نگرانی­های بهداشت عمومی می­باشد. در همین راستا افزایش کیفیت و سال­های زندگی توأم با سلامت از اهداف مهم انسان سالم تا سال 2010 می­باشد. مواد و روش­ها: این مطالعه یک مطالعه مداخله­ای برای ارزشیابی تأثیر مداخله آموزشی در ارتقاء کیفیت زندگی سالمندان ساکن آسایشگاه کهریزک می­باشد. در این مطالعه یک نمونه 60 نفری از سالمندان سنین 60 تا 85 پرسش­نامه SF-36 را قبل و ده هفته پس از مداخله تکمیل نمودند. پایایی پرسش­نامه با استفاده از روش بازآزمایی سنجیده شد. نتایج با استفاده از آزمون t و کای- دو تجزیه و تحلیل شدند. نتایج: بر اساس نتایج مطالعه حاضر، مداخله آموزشی تاًثیر معناداری در تمام ابعاد کیفیت زندگی از قبیل: عملکرد جسمانی (05/0>P)، محدودیت نقش جسمانی (00001/0>P)، درد بدنی (001/0>P)، سلامت عمومی (01/0>P)، نیروی حیاتی (008/0>P)، عملکرد اجتماعی (001/0>P)، محدودیت نقش روانی (00001/0>P) و سلامت روان (03/0>P) داشته است. نتیجه­گیری: برنامه­های آموزش بهداشت تأثیر مثبتی در ارتقاء کیفیت زندگی دارند. بنابراین توصیه می­گردد برنامه آموزش بهداشت را در مداخلات ارتقاء کیفیت زندگی به­کار بندیم.

Title: بررسی تأثیر جلوه‌هاي ويژه¬ي فیلم¬های آموزشی بر میزان یادگیری

http://knh.shmu.ac.ir/index.php/site/article/view/172

مقدمه: از نظر پیاژه کودکان و هم نوجوانان به فیلم­هایی که با تصویر زندگی عادی و روزمره­شان تفاوت داشته باشند و برایشان بدیع و غریب جلوه کند، توجه بیش­تری نشان می­دهند. هدف این پژوهش بررسی تأثیر جلوه‌هاي ويژه­ي فیلم­های آموزشی بر میزان یادگیری می­باشد. مواد و روش­ها: در این پژوهش از میان 1777 دانش­آموز پایه چهارم مدارس دولتی شاهرود به روش نمونه­گیری خوشه­ای تصادفی 8 کلاس (250 دانش­آموز) و از فصول کتاب علوم، 4 فصل انتخاب شده و 8 فیلم در رابطه با محتوای فصول به­صورت با جلوه‌هاي ويژه و بدون جلوه‌هاي ويژه تولید و پس از نمایش فیلم­ها، آزمونی حاوی 20 سؤال بر مبنای محتوای فیلم­های آموزشی گرفته شد (برای هر فصل 5 سؤال). پس از آزمون، میانگین نمرات با استفاده از آزمون t مستقل در دختران و پسران تجزیه و تحلیل شد. نتایج: نتایج نشان داد که میانگین نمرات دانش­آموزان پسری که فیلم با جلوه­های ویژه دیده­اند برابر با 64/3 و در دانش­آموزانی که فیلم بدون جلوه ویژه دیده­اند برابر 65/3 می­باشد که اختلاف آن­ها معنادار نمی­باشد اما میانگین نمرات دانش­آموزان دختری که فیلم دارای جلوه­های ویژه دیده­اند برابر با 52/3 و دانش­آموزان دختری که فیلم بدون جلوه­های ویژه دیده­اند برابر با 29/3 می­باشد، که این اختلاف معنادار است. نتیجه­گیری: خلق فضاهای جذاب در فیلم به کمک تکنیک جلوه­های ویژه امکان می­یابد، که ساده­ترین آن افکت­های تصویری مانند دیزالو، فید و سوپر ایمپوز می­باشد. تکنیک­هایی که در کامپیوترهای شخصی هم به سهولت قابل دسترسی است. امروزه کامپیوتر برای همیشه جامعه و فرایندهای یادگیری را دچار دگرگونی کرده است. در این پژوهش در یادگیری از فیلم­های دارای جلوه­های ویژه، جنسیت تأثیر داشت که این مطلب می­تواند دلالت بر سطوح برانگیختگی بالاتر در پسران به­دلیل مواجهه بیش­تر با انواع بازی­های کامپیوتری و فیلم­های پرتحرک در مقایسه با دختران باشد.

Title: ارزیابی عملکرد رزین نانو ذرات آهن (لیواتیت FO36) در کاهش کروم شش ظرفیتی از محيط مايي

http://knh.shmu.ac.ir/index.php/site/article/view/63

مقدمه: کروم شش ظرفیتی را می­توان به روش­های مختلفی از محیط­های مایی حذف کرد اما غالب این روش­ها هزینه­بر می­باشند. در این مطالعه از رزین تبادل­کننده لیواتیت FO36 به­عنوان یک روش جدید برای حذف کروم استفاده شده است. مواد و روش­ها: حذف یون کروم شش ظرفیتی از محیط­های مایی با رزین تبادل­کننده لیواتیت FO36 در وضعیت­های مختلف از جمله اثر میزان ماده جاذب، غلظت کروم اولیه، زمان تماس و pH در فاز ناپیوسته مورد بررسی و مطالعه قرار گرفت. ثابت تعادل كه به ظرفیت تبادل یون بستگی دارد توسط ایزوترم جذب لانگمیر به­دست آمد. نتایج: در پروسه تبادل­کننده ناپیوسته، زمان تعادل بعد از 90 دقیقه تماس حاصل گردید و میزان حداکثر حذف کروم شش ظرفیتی، در میزان pH 5 تا 8 برای غلظت کروم شش ظرفیتی، 5/0 میلی­گرم در لیتر، به­دست آمد. لیواتیت Fo36 قدرت باند شدن بالایی دارد و میزان حداکثر ظرفیت جذب به­دست آمده 29/0 میلی مول از کروم شش ظرفیتی برای Fo36 در 6= pH  به­دست آمد و اثر تبادل­کنندگی کروم شش ظرفیتی بر روی رزین تبادل­کننده کاتیونی از معادله واکنش درجه اول پیروی کرد. نتیجه­گیری: بنابراين بر اساس يافته­های این مطالعه چنين می­توان گفت که رزین لیواتیت Fo36 می­تواند به­عنوان یک گزینه مناسب با توجه با قابل احیاء بودن آن برای کاهش میزان کروم شش ظرفیتی از محیط­های مایی در مناطق آلوده کشور و به­خصوص خراسان جنوبی که داری مشکل حضور کروم در آب آشامیدنی می­باشد، به­کار رود.

Title: بررسی عملکرد تصفیه‌خانه فاضلاب بیمارستان امام حسین (ع) شاهرود

http://knh.shmu.ac.ir/index.php/site/article/view/181

مقدمه: در اثر فعالیت­های مختلف در بیمارستان، روزانه حجم زیادی فاضلاب تولید می­شود که به­دلیل آلودگی شدید آن باید به روش مناسبی تصفیه گردد. بیمارستان امام حسین (ع) شاهرود نیز دارای تصفیه­خانه فاضلاب از نوع هوادهی گسترده می­باشد. به منظور بررسی عملکرد این تصفیه­خانه و بررسی امکان استفاده مجدد پساب آن برای آبیاری فضای سبز و یا تخلیه در محیط، مطالعه حاضر صورت گرفت. مواد و روش­ها: در این مطالعه مقطعی، عملکرد تصفیه­خانه فاضلاب بیمارستان امام حسین(ع) شاهرود طی مدت 8 ماه، در فاصله اردیبهشت تا آذر 87 با استفاده از پارامترهای: COD، BOD5، TSS، pH، کل کلیفرم­ها و کلیفرم­های مدفوعی در فاضلاب ورودی و خروجی بر اساس روش­های استاندارد تعیین و با استانداردهای سازمان حفاظت محیط زیست ایران با استفاده از آزمون t مقایسه شده است. یافته­ها: مقادیر میانگین  pH،TSS،BOD5 ،COD ، میانگین هندسی کل کلیفرم­ها، میانگین هندسی کلیفرم­های مدفوعی در پسآب به­ترتیب برابر 95/6، mg/l 179، mg/l145، mg/l298،  MPN/100104×8/2 و  MPN/100104×54/1 می­باشد. مقدار حذف آلاینده­ها به­ترتیب 11/46%=TSS، 1/30%=COD، 99/39%=BOD5، 37/99%= کل کلیفرم­ها و کلی فرم­های مدفوعی برابر با 35/99% می­باشد که با استانداردهای سازمان محیط زیست ایران اختلاف معناداری را نشان می­دهند (001/0>P). نتیجه­گیری: در صورت استفاده مجدد از پسآب برای آبیاری فضای سبز و کشاورزی باید کاهش آلودگی شیمیایی و میکروبی در حد استانداردهای سازمان حفاظت محیط زیست ایران مورد توجه قرار گیرد.
